# Daratumumab for the Management of Newly Diagnosed and Relapsed/Refractory Multiple Myeloma: Current and Emerging Treatments

**DOI:** 10.3389/fonc.2020.624661

**Published:** 2021-02-17

**Authors:** Massimo Offidani, Laura Corvatta, Sonia Morè, Davide Nappi, Giovanni Martinelli, Attilio Olivieri, Claudio Cerchione

**Affiliations:** ^1^ Clinica di Ematologia Azienda Ospedaliero-Universitaria, Ospedali Riuniti di Ancona, Ancona, Italy; ^2^ Department of Hematology and Cell Bone Marrow Transplantation (CBMT), Ospedale di Bolzano, Bolzano, Italy; ^3^ Hematology Unit, Istituto Scientifico Romagnolo per lo Studio e la Cura dei Tumori (IRST) Istituto di Ricovero e Cura a Carattere Scientifico (IRCCS), Meldola, Italy

**Keywords:** daratumumab, multiple myeloma, relapsed refractory multiple myeloma, newly diagnosed multiple myeloma, anti CD38

## Abstract

Immunotherapy is changing the paradigm of multiple myeloma (MM) management and daratumumab is the first-in-class human monoclonal antibody targeting CD38 approved for the treatment of this malignancy. Daratumumab exerts anti-myeloma activity by different mechanisms of action as antibody-dependent cellular cytotoxicity (ADCC), antibody-dependent cellular phagocytosis (ADCP), complement-dependent cytotoxicity (CDC), direct apoptosis, and immunomodulation. After GEN501 and SIRIUS trials showed efficacy of daratumumab monotherapy in heavily pretreated relapsed-refractory multiple myeloma (RRMM), in patients with at least two previous line of therapy, two phase III trials demonstrated superior overall response rate (ORR) and progression free survival (PFS) using triplets daratumumab–bortezomib–dexamethasone (DVd) *vs* Vd (CASTOR) or daratumumab–lenalidomide–dexamethasone (DRd) *vs* Rd (POLLUX) in relapsed-refractory MM patients; so these combinations have been approved and introduced in clinical practice. The ongoing phase III CANDOR is evaluating the triplet daratumumab–carfilzomib–dexamethasone (DKd) *vs* Kd whereas phase III APOLLO trial is exploring daratumumab–pomalidomide–dexamethasone (DPd) *vs* PD. Many other trials exploring daratumumab combinations in relapsed-refractory MM are ongoing, and they will provide other interesting results. In newly diagnosed transplant-eligible patients, phase III CASSIOPEIA trial found the combination daratumumab–bortezomib–thalidomide–dexamethasone (Dara-VTd) significantly improves stringent Complete Response (sCR) rate and PFS compared with VTD, whereas in the phase II GRIFFIN study, comparing daratumumab–bortezomib–lenalidomide–dexamethasone (Dara-VRD) *vs* VRD, sCR rate was significantly higher using quadruplet combination. Many studies are evaluating daratumumab in consolidation and maintenance therapy after autologous stem cell transplantation (ASCT). As regard patients ineligible for ASCT, a great efficacy of daratumumab-containing combinations was reported by the phase III trials ALCYONE and MAIA, exploring daratumumab–bortezomib–melphalan–prednisone (DVMP) *vs* VMP and daratumumab–lenalidomide–dexamethasone (DRd) *vs* Rd, respectively. These studies provided results never seen before in this setting. The aim of this paper is to critically review the results obtained with regimens containing daratumumab both in relapsed-refractory and in newly diagnosed MM.

## Introduction

Immunotherapy is changing the paradigm of MM management and daratumumab is the first-in-class human monoclonal antibody targeting CD38 approved for the treatment of this malignancy. Daratumumab exerts anti-myeloma activity by different mechanisms of action as antibody-dependent cellular cytotoxicity (ADCC), antibody-dependent cellular phagocytosis (ADCP), complement-dependent cytotoxicity (CDC), direct apoptosis and immunomodulation. After the GEN501 and SIRIUS trials showed efficacy of daratumumab monotherapy in heavily pretreated RRMM, in patients with at least two previous lines of therapy, two phase III trials demonstrated superior ORR and PFS using triplets daratumumab–bortezomib–dexamethasone (DVd) *vs* Vd (CASTOR) or daratumumab–lenalidomide–dexamethasone (DRd) *vs* Rd (POLLUX) in relapsed-refractory MM patients; so these combinations have been approved and introduced in clinical practice. The ongoing phase III CANDOR is evaluating the triplet daratumumab–carfilzomib–dexamethasone (DKd) *vs* Kd whereas phase III APOLLO trial is exploring daratumumab–pomalidomide–dexamethasone (DPd) *vs* PD. Many other trials exploring daratumumab combinations in relapsed-refractory MM are ongoing, and they will provide other interesting results.

In newly diagnosed transplant-eligible patients, phase III CASSIOPEIA trial found the combination daratumumab–bortezomib–thalidomide–dexamethasone (Dara-VTd) significantly improves sCR rate and PFS compared with VTD, whereas in the phase II GRIFFIN study, comparing daratumumab–bortezomib–lenalidomide–dexamethasone (Dara-VRD) *vs* VRD, sCR rate was significantly higher using quadruplet combination. Many studies are evaluating daratumumab in consolidation and maintenance therapy after ASCT. As regard patients ineligible for ASCT, a great efficacy of daratumumab-containing combinations was reported by the phase III trials ALCYONE and MAIA exploring daratumumab–bortezomib–melphalan–prednisone (DVMP) *vs* VMP and daratumumab–lenalidomide–dexamethasone (DRd) *vs* Rd, respectively. These studies provided results never seen before in this setting.

## Transplant-Eligible Newly Diagnosed Multiple Myeloma Patients

In young MM patients, ten-year survival increased from 18% in 2002–2006 to 35% in 2012–2016 (1), and this improvement has been related to the growing number of available therapeutic options since the 2000s. Previously, the development of autologous stem cell transplantation (ASCT) in the 1990s (2) had already contributed to a significantly increased survival, and now triplet novel agent regimens followed by ASCT represent the standard treatment for eligible patients. This therapeutic approach led to a ten-year survival of 60% (3). In Europe bortezomib, thalidomide dexamethasone (VTD) and bortezomib, cyclophosphamide, dexamethasone (VCD) combinations represent the most used regimens as induction therapy before ASCT, whereas in the USA bortezomib, lenalidomide, dexamethasone (VRD) is the preferred regimen according to the latest National Comprehensive Cancer Network (NCCN) guidelines.

The impact of daratumumab in combination with VTd (D-VTd) *vs* VTd as induction and consolidation therapy post ASCT was assessed in the phase III CASSIOPEIA trial (4) including 1,085 patients enrolled in 111 European sites. The primary endpoint of the study was the rate of sCR after consolidation, whereas secondary goals were minimal residual disease (MRD) negativity and ≥CR rates, PFS, and OS. Patients were randomized to four induction cycles and two consolidation cycles with VTd including bortezomib (1.3 mg/m^2^ on days 1, 4, 8, 11), thalidomide (100 mg daily), and dexamethasone or D-VTd with intravenously daratumumab at a dose of 16 mg/kg once weekly in induction cycles 1 and 2 and once every 2 weeks during all the other cycles. Patients achieving at least a PR at day 100 post-ASCT were further randomized to observation or maintenance therapy with daratumumab every 8 weeks for 2 years. Median age of patients receiving VTd and D-VTd were 58 and 59 years and high-risk cytogenetics were documented in 16 and 15% of patients, respectively. Stringent CR rate after consolidation was significantly better in the D-VTd group compared with VTd (29 *vs* 20%; p = 0.0010), and this superiority was consistent across all subgroups of patients except for those with high-risk cytogenetics and ISS stage III (4). As regard MRD status after consolidation, a higher proportion of patients treated with D-VTd achieved MRD negativity assessed by multiparametric flow cytometry (MFC, 10^−5^) (64 *vs* 44%; p <0.0001), and this benefit was documented also in high-risk cytogenetics (60 *vs* 44%, OR = 1.88) and ISS stage III (64 *vs* 46%, OR = 2.14) subgroups (5). The assessment of MRD status with next-generation sequencing (NGS, 10^−6^) showed a negativity in 39% of patients receiving D-VTd *vs* 23% VTd, (p < 0.0001) (6). In the CASSIOPET companion study (7) including 268 patients enrolled in CASSIOPEIA trial, more patients with a response ≥CR receiving D-VTd *vs* VTd achieved PET/CT and MRD double negativity after consolidation (41.7 *vs* 25%; p = 0.0206). Regarding survival measures, PFS at 18 months was 93% in the D-VTd group *vs* 85% in the VTd group (HR = 0.47; p < 0.0001), whereas the short median follow-up (18.8 months) makes survival data immature. However, it should be outlined that no maintenance was planned in the VTd arm, and lenalidomide maintenance, actually a standard therapy post-ASCT, could have prolonged PFS in patients enrolled in the standard arm. Overall, toxicity was not increased when adding daratumumab to VTd, and the most common grade 3–4 side effect was neutropenia occurring in 28 and 15% of patients treated with D-VTd and VTd, respectively. Among non-hematologic toxicities, grade 3–4 infections occurred in 22% of D-VTd patients *vs* 20% in VTd whereas grade 3–4 peripheral neuropathy developed in 9% of both groups. The rate of treatment discontinuation due to side effects was 7% in the D-VTd group and 8% in VTd (4). Of note, a comparison between patients with baseline conventional “CRAB” diagnostic criteria and those with “slim” only criteria showed no significant differences in terms of response rates, MRD-negativity rates, and PFS (8). Based on these results, both FDA and EMA regulatory agencies approved D-VTd in the early 2020.

No randomized trials have directly compared D-VTd to VRD but a matching-adjusted indirect comparison (MAIC) of 543 patients receiving four courses of D-VTd plus ASCT *vs* 350 patients enrolled in the IFM2009 trial and treated with three cycles VRD plus ASCT, has been presented at the last International Myeloma Workshop (9). This MAIC showed that D-VTd plus ASCT significantly improves PFS and MRD negativity compared to VRD plus ASCT.

Lenalidomide, instead of thalidomide, in combination with bortezomib, dexamethasone and daratumumab (D-VRd) has been evaluated in the randomized phase II GRIFFIN trial (10) whose primary endpoint was the sCR rate by the end of post-ASCT consolidation. All patients were assigned to receive four induction cycles, ASCT and two consolidation cycles with VRd (bortezomib 1.3 mg/m^2^ on days 1, 4, 8, 11; lenalidomide 25 mg daily on days 1–14; dexamethasone 20 mg on days 12, 2, 8, 9, 15, 16) or D-VRd (VRD plus daratumumab 16 mg/kg on days 1, 8, 15 in the induction cycles and on day 1 in the consolidation cycles). After consolidation, maintenance therapy until progression or up to 2 years consisted in lenalidomide for the VRd group and lenalidomide plus daratumumab in the D-VRd group. Among 103 patients receiving VRd and 104 treated with D-VRd, 14 and 16%, respectively, were at high-risk cytogenetics. A sCR post consolidation was achieved in 42.4 and 32% of D-VRd and VRd patients, respectively (p = 0.068, statistically significant at the preset one-sided *α* of 0.10). However, achievement of sCR after consolidation could be debatable as primary endpoint considering that it was foregone that a quadruplet combination including daratumumab, mostly well tolerated, would have resulted in higher response rates than triplet combinations. MRD status would have represented a more significant primary endpoint being a surrogate biomarker for PFS. As regard MRD negativity (10^−5^ threshold), it resulted in 51% in the D-VRd group *vs* 20.4% in the VRd at the last follow-up. Responses deepened over time in both groups of patients, the rate of D-VRd patients with a sCR being 62.6 *vs* 45.4% of VRd patients after a median follow-up of 22.1 months. However, it has to be outlined that a lower percentage of patients receiving D-VRd underwent ASCT (90.4 *vs* 75.7%). Median PFS was not reached in either study arm, but it is presumable that follow-up is too short for detecting a significant difference. As regard toxicity, the most common grade 3–4 side effect was neutropenia (D-VRd 41.4%; VRd 21.6%) whereas the incidence of grade 3–4 infections was 23.2 *vs* 21.6%. The ongoing phase III PERSEUS trial, a collaborative study with European Myeloma Network (EMN) (NCT03710603) with the same study design of GRIFFIN, is evaluating D-VRd (with daratumumab administered subcutaneously) *vs* VRd in 690 patients. The results are awaited since, if efficacy of D-VRd is confirmed, another therapy for patients eligible for ASCT will be available in the future. Another ongoing phase III study (CEPHEUS, NCT03652064) is assessing D-VRd *vs* VRd in patients of all age for whom transplant is not intended as initial therapy, and it will probably provide some answers about the role of ASCT as frontline therapy.

Very important results have been recently reported with the triplet KRd in which a second generation proteasome inhibitor as carfilzomib replaces bortezomib. In a phase II study (11) including 76 patients receiving four cycles of KRd as induction, ASCT, four cycles KRd as consolidation, and 10 cycles of KRd as maintenance, after consolidation 90% of patients achieved at least a VGPR, 60% sCR and 61% MRD negativity assessed by next generation sequencing (NGS) with <10^−5^ sensitivity. After a median follow-up of 56 months, 5-year PFS and OS were 72 and 84%, respectively. These results are similar to those reported by the phase II randomized FORTE trial (12, 13) in which 474 newly diagnosed MM patients were randomized to receive four KRd induction cycles, ASCT, four consolidation KRd cycles; 12 KRd cycles or four KCd induction cycles, ASCT, four KCd consolidation cycles. The rates of post consolidation ≥VGPR, ≥CR, and MRD negativity (at a cut-off of at least 10^−5^) were 89, 60, and 58%, respectively. The addition of daratumumab to KRd was found to be tolerated in a phase 1b study (NCT01998971) (14) including newly diagnosed MM patients regardless of transplant eligibility. Patients received a median of 11 cycles of quadruplet D-KRd that yielded an ORR of 100% with 91% of patients achieving at least VGPR and 43% a CR. Based on these results, a phase II study in which 24 cycles of D-KRd is administered as initial therapy for patients of all ages is ongoing (NCT 03500445).

A phase II study of KRd-D with carfilzomib administered weekly (20 mg/m^2^ on day 1 of cycle 1, 56 mg/m^2^ on days 8 and 15 of cycle 1 and days 1, 8, and 15 of cycles 2–8) for eight cycles has been presented at the last American Society of Hematology (ASH) (15). Peripheral stem cell collection was recommended after four to six cycles of therapy for eligible patients but wKRd-D was continued for a total of eight cycles. Thirty patients with a median age of 57 years (range 36–70 years) were enrolled. MRD negativity rate (at level of 10^−5^), the primary endpoint of study, was 75% in the 24 patients who completed eight cycles (ORR = 100%; ≥VGPR = 92%). These data are very interesting considering that 49% of the patients were at high-risk cytogenetics. The phase III ADVANCE trial (NCT04268498) is evaluating wKRd-D *vs* wKRd and VsRD.

In the ongoing phase II MASTER trial (16) 101 patients received four cycles of D-KRd (daratumumab 16 mg/kg on days 1, 8, 15, and 22 of cycles 1 and 2 and less frequently in the subsequent cycles; carfilzomib 56 mg/m^2^ on days 1, 8, and 15; lenalidomide 25 mg days 1–21; dexamethasone 40 mg on days 1, 8, 15, and 22) as induction, ASCT and 0, 4, or 8 cycles of D-KRd consolidation according to MRD status evaluated by NGS assay (<10^−5^) at each phase of therapy. Patients who received therapy until two consecutive assessments were negative for MRD status, whereas patients who were MRD positive at the end of consolidation received lenalidomide maintenance. MRD negativity rate was 42% post induction, 73% post ASCT, and 82% during consolidation MRD-adapted. Of note, MRD negativity rates were similar between the standard and the high-risk cytogenetic groups. Most common grade 3–4 side effects were neutropenia (25%) and infections (12%).

An all oral regimen with ixazomib, the first approved oral proteasome inhibitor, lenalidomide and dexamethasone, has been assessed in combination with daratumumab in a phase II study (17) including MM patients irrespective of their transplant eligibility. Treatment consisted of 12 cycles with D-IxaRd as induction (ixazomib 4 mg days 1, 8, 15; lenalidomide 25 mg days 1–21; dexamethasone 40 mg weekly and daratumumab 16 mg/kg weekly for two cycles, every other week during cycles 3–6 and every 4 weeks afterwards) followed by 24 courses of daratumumab 16 mg/kg every 4 weeks plus ixazomib on days 1, 8, and 15 as maintenance. In patients who were ASCT eligible, stem cells were collected after four D-IxaRd cycles. The median age of 40 enrolled patients was 64.5 years (range 33–81 years). After a median follow-up of 10.1 months, response rates ≥VGPR and ≥CR were documented in 69 and 19% of patients, respectively, with 28% achieving MRD negativity. Treatment was well tolerated and the main toxicities were grade 3–4 neutropenia occurring in 16% of patients and infections in 3%. Rash developed in 48% of patients, but it was mainly of grades 1–2 (45%).

The triplet VCD, as mentioned above, represents another regimen frequently used as induction in patients eligible for ASCT, although a phase III trial (18) and a retrospective analysis by GIMEMA and European Myeloma Network (EMN) (19) reported a higher quality of response with VTD than VCD. However, as well as for the other triplets, also VCD has been evaluated in combination with daratumumab. In the phase II LYRA study (20) 86 patients, irrespective of eligibility for ASCT, (median age 63, range 41–82 years; 37% with high-risk cytogenetics) received 4–8 cycles of induction therapy with bortezomib 1.5 mg/m^2^ on days 1, 8, and 15; oral cyclophosphamide 300 mg/m^2^ on days 1, 8, 15, and 22; dexamethasone 40 mg weekly and daratumumab 16 mg/kg weekly for two cycles, every two weeks for four cycles and every 4 weeks for the last two cycles. After induction patients could receive ASCT at the discretion of the investigator and afterwards a maintenance with monthly daratumumab for 12 cycles. The rate of CR + VGPR after four cycles (the primary endpoint) was 44% with an ORR of 79%. The same combination was assessed in a phase 1b study (21) in which 18 patients received four induction cycles with CyBorD-Dara, ASCT, two consolidation cycles with CyBorD-Dara, and a maintenance with daratumumab every 4 weeks until progression. Overall, treatment was safe, and 94 and 44% of patients, respectively, achieved at least VGPR and CR after ASCT. Remarkably, 44% of patients obtained MRD negativity at a level of 10^−5^ after consolidation. The ongoing phase II randomized trial EMN 18 (NCT03896737) is testing a therapeutic strategy including four cycles of Dara-VCD as induction, ASCT, four cycles of Dara-VCD as consolidation *vs* four VTd, ASCT, four VTd. At the end of the consolidation the patients are randomized to receive a maintenance with daratumumab alone or plus ixazomib for up 24 months.

**Figure 1:**
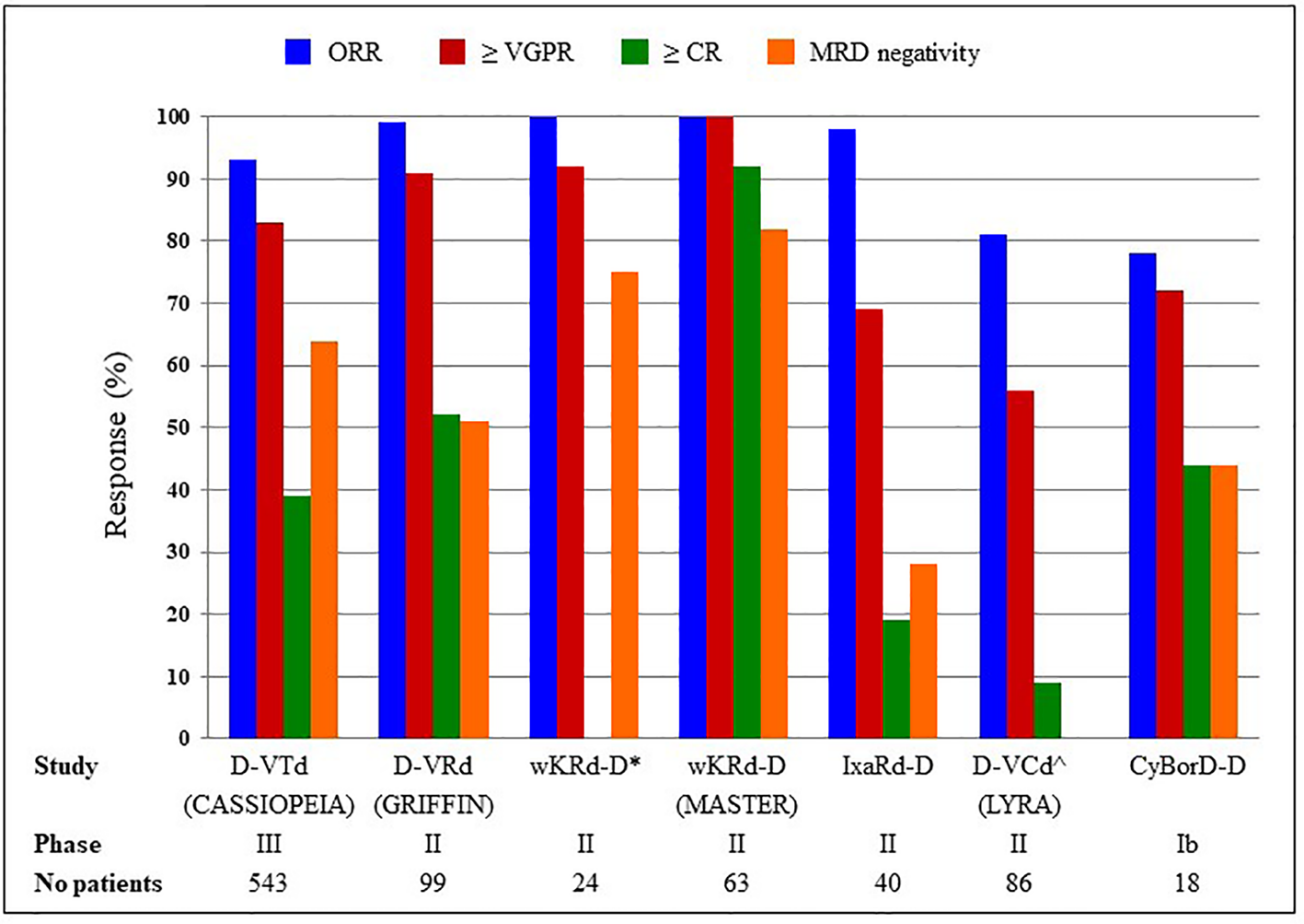
D-VTd, daratumumab, bortezomib, thalidomide, dexamethasone; D-VRd, daratumumab, bortezomib, lenelidomide, dexamethasone; wKRd_D, weekly carfilzomib, lenalidomide, dexamethasone, daratumumab; IxaRd-D, ixazomib, lenalidomide, dexamethasone, daratumumab; D-VCd and CyBorD-D, daratumumab, cyclophosphamide, bortezomib, dexamethasone. *≥ CR not available; ^MRD status not available.

After a meta-analysis (22) confirmed the advantage in terms of PFS and OS of lenalidomide maintenance post-ASCT, several trials are evaluating daratumumab alone or in combination with other agents in this setting. In **Figure 1** we reported results of the main daratumumab-containing combinations in MM patients eligible for ASCT, whereas in **Table 1** we summarized other not mentioned ongoing clinical trials.

**Table 1:** Ongoing clinical trial with daratumumab in transplant eligible newly diagnosed MM patients.

Trial	Phase	Characteristics of patients	Design	ClinicalTrials N.
MUK Nine b: OPTIMUM Treatment Protocol (MUKnineb)	II	Transplant eligible with high-risk NDMM and plasma cell leukemia	CVRDd × 4-6 (induction)→SCT→DVRd x 6 (consolidation part 1) → DVR × 12 (consolidation part 2)→DR (maintenance)	03188172
Study association of lenalidomide, ixazomib, dexamethasone and daratumumab in newly diagnosed standard risk multiple myeloma (IFM2018-01)	II	Transplant eligible with standard-risk NDMM	IxaRd-D (induction) → SCT → IxaRdD (consolidation) → R (maintenance)	03669445
Daratumumab, carfilzomib, lenalidomide, and low dose dexamethasone (DKRd) in newly diagnosed, multiple myeloma	II	Transplant and non-transplant eligible NDMM	DKRd x 24 cycles	03500445
Ixazomib citrate, lenalidomide, dexamethasone, and daratumumabintreating patients with newly diagnosed multiple myeloma	II	Transplant and non-transplant eligible NDMM	IxaRdD × 12 (induction) → IxaD for up to 36 months (maintenance)	03012880
Daratumumab, ixazomib, and dexamethasone or daratumumab, bortezomib, and dexamethasone in patients with newly diagnosed multiple myeloma (DeRIVE)	II	Transplant and non-transplant eligible NDMM	Arm 1: IxaDd x 8 (induction) → ± SCT → IxaDd for up to 24 months (maintenance) Arm 2: DVd x 3 followed by IxaDd x 5 (induction) → ± SCT → IxaDd for up to 24 months (maintenance)	03944224
An intensive program with with quadruplet induction and consolidation plus tandem autologous stem cell transplantation in newly diagnosed high-risk multiple myeloma patients (IFM 2018-04)	II	Transplant eligible with high-risk NDMM	DKRd × 6 (induction) → tandem SCT → DKRd × 4 (consolidation) → DR (maintenance)	03606577
Study of daratumumab combined with carfilzomib, lenalidomide, and dexamethasone for newly diagnosed multiple myeloma	II	Transplant and non-transplant eligible NDMM	DKRd x 8 (induction) → MRD based therapy (post-induction)	04113018
2015-12: A study exploring the use of early and late consolidation/maintenance therapy	II	Transplant eligible with high-risk NDMM	DKTd-PACE ×→ SCT → DKd ± SCT (consolidation 1) → D (consolidation 2) → DKd alternating with DRd in 3-month blocks	03004287
Adaptive strategy in treatment for newly diagnosed multiple myeloma with upfront daratumumab-based therapy	II	Transplant and non-transplant eligible NDMM	DRd (induction) → DVRd (consolidation MRD based) → DR→R (maintenance)	04140162
Daratumumab in treating transplant-eligible partecipants with multiple myeloma	II	Transplant eligible with NDMM who have received any prior induction therapy	D × 2 (consolidation 1) → SCT (consolidation 2) → DR × 12→D (maintenance)	03477539
Short course daratumumab in patients with multiple myeloma	II	Transplant with NDMM who have achieved VGPR or better after induction ± consolidation/SCT	DR × 6 months	03490344
A study of daratumumab plus lenalidomide *versus* lenalidomide alone as maintenance treatment in participants with newly diagnosed multiple myeloma who are minimal residual disease positive after frontline ASCT (AURIGA)	III	Transplant eligible with NDMM who have received induction ± consolidation and SCT	DR *vs* R until progression	03901963
S1803, daratumumab/rHuPh20+/− lenalidomide as post-ASCT maintenance for MM w/MRD to direct therapy duration (DRAMATIC)	III	Transplant eligible with NDMM who have received induction and SCT	DR *vs* R, duration guided by MRD status	04071457

C, cyclophosphamide; V, bortezomib; R, lenalidomide; D, daratumumab; d, dexamethasone; Ixa, ixazomib; K, carfilzomib; T, thalidomide; SCT, stem cell transplant.

## Transplant-Ineligible Newly Diagnosed Multiple Myeloma Patients

Daratumumab recently has obtained good results also in the setting of newly diagnosed MM patients not eligible for ASCT since it has been approved in combination with bortezomib–melphalan–dexamethasone (D-VMP) and with lenalidomide–dexamethasone (D-Rd).

In the phase III trial ALCYONE (23, 24) 706 patients (median age 71 years) were randomized to receive nine cycles with VMP (bortezomib 1.3 mg/m^2^ twice weekly in cycle 1 and once weekly in cycles 2–9; melphalan 9 mg/m^2^ on days 1–4 and dexamethasone 60 mg/m^2^ on days 1–4) or D-VMP (the same schedule of VMP plus daratumumab 16 mg/kg weekly in cycle 1, every 3 weeks in cycles 2–9 and every 4 weeks subsequently). After a median follow-up of 40.1 months median PFS, the primary endpoint of the study was 36.4 in the D-VMP group *vs* 19.3 in the VMP group (HR = 0.42; p < 0.0001). Remarkably, PFS curve of VMP arm starts to show a much higher slope if compared with D-VMP curve just after nine cycles, emphasizing the benefit of daratumumab maintenance *vs* fixed duration therapy. The lack of maintenance in the VMP arm also explains such a high difference (58%) in the risk of progression/death between the two regimens.

The superiority of D-VMP was consistent across all subgroups of patients including those older than 75 years or with ISS stage III, whereas hazard ratio was lower in patients with standard-risk *vs* high-risk cytogenetics (0.39 *vs* 0.78). Overall, after a longer follow-up, a benefit for OS was also observed since patients receiving D-VMP showed a 40% reduction in the risk of death with an estimated 42-month OS rate of 75% with D-VMP *vs* 62% with VMP (HR = 0.60; p = 0.0003). The D-VMP group had higher overall response rates (91 *vs* 74%; p < 0.0001), ≥CR rate (46 *vs* 25%; p < 0.0001), and MRD negativity rate (28 *vs* 7%; p < 0.0001) compared with the VMP group. Remarkably, in a subgroup analysis a sustained MRD negativity for at least 12 months *vs <*12 months was associated with better outcomes in terms of PFS and OS (24). As for safety, infections were most commonly reported in grade 3–4 adverse event (23% in the D-VMP group and 14.7% in the VMP group), particularly pneumonia (11.3 *vs* 4%). In addition, fewer daratumumab-treated patients discontinued treatment due to adverse events, compared with the VMP-treated patients (5 *vs* 9%). During daratumumab monotherapy in the D-VMP group, the most frequent any grade adverse events were upper respiratory infections (19%) and bronchitis (15%). Simultaneously, the phase III trial MAIA (25, 26) compared the standard of care lenalidomide–dexamethasone (Rd) with Rd plus daratumumab (D-Rd) in 737 newly diagnosed MM patients with a median age of 73 years. Patients enrolled in the Rd arm received lenalidomide 25 mg on days 1–21 plus dexamethasone 40 mg on days 1, 8, 15 and 22; patients allocated in the D-Rd arm were treated with Rd plus daratumumab at a dose of 16 mg/kg once weekly during cycles 1,2, every two weeks during cycles 3–6 and every 4 weeks thereafter. Treatment was continued until progression or unacceptable toxicity. In the last update of the study, after a median follow-up of 36.4 months, D-Rd demonstrated a significant PFS benefit since median PFS was not reached *vs* 33.8 months in D-Rd and Rd groups, respectively (HR = 0.56; p < 0.0001). Of note, 36-months PFS was 68% in the D-Rd group *vs* 46% in the Rd group. Although comparison between different trials should be made with caution, median PFS of the population treated with Rd in the MAIA trial is quite similar to that treated with Dara-VMP in the ALCYONE one (33.8 *vs* 36 months). This further outlines the better performance of Dara-Rd compared with Dara-VMP despite the higher HR (0.56 *vs* 0.42). The ORR was 93 *vs* 82%, with CR rates or better 50 *vs* 27% (p < 0.0001), respectively. MRD negativity was also significantly more frequent in patients treated with D-Rd *vs* Rd, being 29 *vs* 9% (p < 0.0001).

In the forest plot for PFS, D-Rd turned out to be favorable in all subgroups, but its benefit seemed less strong in the high-risk cytogenetic compared to the standard-risk patients (HR 0.52 *vs* 0.49). The most frequent grade 3–4 hematological adverse event reported in the study was neutropenia (50% in the D-Rd group *vs* 35.3% in the Rd group) whereas among non-hematologic toxicities grade 3–4 infections developed in 32 and 23.3% of D-Rd and Rd patients, respectively, with pneumonia occurring in 13.7 *vs* 8%. The addition of daratumumab to Rd did not increase the incidence of second primary malignancies (8.8 *vs* 7.1%).

D-VMP and D-Rd are actually recommended by the NCCN Guidelines as the preferred Category 1 therapeutic options for newly diagnosed MM patients not eligible for ASCT. However, considering that infections, mainly pneumonia, represent a frequent adverse event in patients receiving Dara-VMP and D-Rd, a recent pooled retrospective analysis of ALCYONE and MAIA trials assessed predictive markers of grade ≥3 and serious infection occurring during the first 6 months of treatment. Using four parameters (age, LDH, albumin, and baseline ALT) patients were classified as low- and high-risk with infection rates of 15.7 and 29.3%, respectively (HR = 2.11; p = 0.0001) (27).

Recently, the PEGASUS study (28) made an anchored indirect treatment comparison (ITC) in terms of PFS among patients treated with D-Rd in the MAIA trial and those receiving VRd or Vd in real life. This analysis demonstrated that D-Rd reduced the risk of progression or death compared to either VRd (HR 0.68; p = 0.04) or Vd (HR 0.48; p < 0.001) in transplant-ineligible patients.

Daratumumab was also studied in association with ixazomib and low dose dexamethasone in phase II HOVON-143 trial (29) for unfit and frail patients according to the International Myeloma Working Group Frailty Index (IMWG-FI). Treatment consisted of nine cycles with ixazomib (4 mg on days 1, 8, 15), daratumumab (16 mg/kg weekly cycles 1 and 2; every two weeks cycles 3–6; day 1 cycles 7–9), dexamethasone (in combinations with daratumumab 10 mg). Maintenance therapy until progression or for maximum of 2 years included daratumumab plus ixazomib. Results of the 65 frail patients enrolled in the study (median age 81 years, range 70–92 years) have been recently presented at the last European Hematology Association (EHA) Congress (30). Overall response rate, primary endpoint, was 78% with 36% of the patients achieving at least a VGPR. However, 12 patients (15%) died due to toxicity, and among them six died early (≤60 days). After a median follow-up of 16.3 months, median PFS was 13.8 months, and 1-year OS was 78%.

Several ongoing phase II and III studies are assessing daratumumab-based combinations in elderly patients. In a phase II US study (NCT 04052880), patients older than 70 years receive subcutaneous daratumumab, dose-attenuated bortezomib, lenalidomide, and dexamethasone until progression with the aim to evaluate response VGPR or better after 8 cycles. In another phase II study (NCT04151667), patients 65 years and older are treated with a response adapted approach, receiving subcutaneous daratumumab plus dexamethasone for 2 months and a subsequent therapy according to response. A phase II randomized clinical trial (NCT04009109) will evaluate 12 cycles with ixazomib plus D-Rd followed by either lenalidomide maintenance or maintenance with lenalidomide, ixazomib, daratumumab for at least 2 years. The IFM 2017-03 phase III trial (NCT03993912) compares subcutaneous daratumumab associated with lenalidomide to Rd until progression in frail patients. Another phase III trial by PETHEMA group (NCT03742297) enrolling elderly fit patients aged between 65 and 80 years randomizes patients to nine cycles VMP followed by nine Rd *vs* 18 cycles KRd *vs* 18 cycles D-KRd.

As regard the key question whether daratumumab is able to improve outcome in patients with high-risk cytogenetics, a recent meta-analysis analyzed six randomized phase III trials, three for newly diagnosed MM (ALCYONE, MAIA, CASSIOPEIA) and three for relapsed/refractory MM (CASTOR, POLLUX, CANDOR). The addition of daratumumab to backbone regimens led to improved PFS among patients with high-risk newly diagnosed MM (pooled HR = 0.67; p = 0.02). However, hazard ratio was better (0.45, p < 0.01) in patients with standard-risk cytogenetics (31).

## Daratumumab in Smoldering Multiple Myeloma

Smoldering multiple myeloma (SMM) represents a very heterogeneous entity, and the question whether patients with SMM should be treated or not remains unresolved. Since the risk of progression for this disease is not uniform over time (32), several studies have been conducted with the aim of recognizing predictive factors and thus of evaluating the risk of progression (33–35). The last risk model by Mayo Clinic (36) categorizes patients in low risk (0 factor), intermediate risk (1 factor) or high risk (2–3 factors) using as risk factors bone marrow plasma cells >20%, serum monoclonal protein >2 g/dl and an involved to uninvolved serum-free light chain ratio >20 (20/2/20 model). The median TTP for low-, intermediate-, and high-risk groups were 110, 68, and 29 months, respectively (p < 0.0001). Current therapeutic approach in patients with smoldering myeloma (SMM) is active monitoring until progression to MM, but different treatments favoring disease control or disease eradication have been evaluated in several studies (37–39), and they are under investigation in other ongoing clinical trials. Based on activity and safety of daratumumab monotherapy in relapsed refractory MM (40), Landgren et al. recently reported results of a randomized, multicenter, phase II study (CENTAURUS) (41) including 123 patients with high or intermediate risk SMM who were randomized to receive three different daratumumab dosing schedules (intense, intermediate, and short). The co-primary endpoint of CR rate >15% was not met since CR rate was lower in all arms of the study, whereas the other co-primary end point of a median PFS ≥24 months in all arms was met. Of note, the 24-month PFS rates were 90, 82, and 75% in the intense, intermediate, and short arm, respectively. The ongoing phase III AQUILA trial (NCT03301220), testing subcutaneous daratumumab *vs* active monitoring will provide further data regarding the efficacy of daratumumab alone in SMM. Another phase II study (NCT03236428) is evaluating daratumumab monotherapy in patients with high-risk MGUS and low-risk SMM with the aim to determine if this agent is able to prevent MM development. In MM setting, daratumumab has also been evaluated in combination with lenalidomde and proteasome inhibitors also in SMM. In the phase II ASCENT study (NCT03289299) high-risk MM patients receive six cycles of induction with D-KRd followed by six consolidation cycles with the same regimen and a maintenance therapy with daratumumab plus lenalidomide for 12 months. Finally, the phase III DETER-MM (NCT 03937635) is assessing, in high-risk SMM, DRd *vs* Rd for 24 cycles.

However, it was emphasized that, at now, no reliable predictive markers of evolution of SMM in overt MM are available. Therefore, we cannot exclude that a not negligible portion of SMM patients treated with the above mentioned trials would never progress to MM.

### Relapsed/Refractory Multiple Myeloma

Daratumumab-based three-drug regimens or as single-agent are treatment options highly efficacious in relapsed/refractory multiple myeloma (RRMM). As described in detail below, several combination treatment strategies with daratumumab, able to prolong PFS when administered until progression are now approved. Combinations with lenalidomide–dexamethasone (DRd) or bortezomid–dexamethasone (DVd) were both firstly approved by both the FDA and EMA. More recently, also combinations with carfilzomib–dexamethasone (DKd) and pomalidomide-dexamethasone (DPd) were approved by the FDA. Single-agent use is labeled for patient refractory to previous lines proteasome inhibitors and immunomodulating-containing agents.

In **Table 2** we summarized the main clinical trials in relapsed/refractory setting.

**Table 2:** Major ongoing clinical trial with daratumumab in refractory/relapsed MM patients.

Trial	Phase	Characteristcs of pts.	Design	ClinicalTrials N
PLEIADES (MMY2040): non-randomized trial exploring daratumumab in combination with various treatment regimens, including Rd	II	RRMM patients ≥1 prior treatment line	RRMM patients received DRd in 28 day-cycle until PD or intolerable toxicity	NCT03412565
MMY1001 trial exploring daratumumab when administered in combination with various treatment regimens, including Kd	Ib	RRMM patients 1–3 prior lines of therapy) carfilzomib-naïve;	RRMM patients received DKd in 28 day-cycle until PD or intolerable toxicity	NCT01998971
CANDOR randomized trial evaluating DKd *vs* Kd in RRMM patients	III	RRMM; 1–3 prior therapies with ≥PR to ≥1 prior therapy	DKd *vs* Kd in 28 day-cycle until PD or intolerable toxicity	NCT03158688
LYNX (MMY2065): randomized trial evaluating DKd *versus* Kd, also for daratumumab-exposed patients	II	RRMM who have received 1–2 prior lines of therapy, daratumumab included	DKd *vs* Kd in 28 day-cycle until PD or intolerable toxicity	NCT03871829
MMY1001trial exploring daratumumab when administered in combination with various treatment regimen, including pomalidomide	IIb	RRMM patients ≥2 prior lines, including V and R	RRMM patients received DPd in 28 day-cycle until PD or intolerable toxicity	NCT01998971
APOLLO: randomized trial evaluating daratumumab + Pd *vs* Pd	III	RRMM ≥1 prior treatment with both lenalidomide and a PI	DPd *vs* PD in 28 day-cycle until PD or intolerable toxicity	NCT03180736
MM-014 non-randomized trial evaluating DPd and Pd in RRMM	II	RRMM patients 1 or 2 prior lines of therapy, including lenalidomide	RRMM patients received DPd in 28 day-cycle until PD or intolerable toxicity	NCT01946477
Randomized trial evaluating daratumumab, cyclophosphamide, dexamethasone plus or not pomalidomide in RRMM	II	RRMM patients ≥1 prior treatment line	Arm A (DCdP) *vs* arm B (DCd plus P, if progressive disease)	NCT03215524
LYRA single arm trial evaluating daratumumab + CyBorD in MM patients, including RRMM	II	RRMM patients 1 treatment line	RRMM patients received DCyborD until progression	NCT02951819
Non-randomized 2-parts trial evaluating venetoclax and daratumumab–dexamethasone plus or not bortezomib i	I/II	RRMM patients with (part-1) or regardless t(11;14) (part-2)	Part-1: VenDd in patients RRMM ≥1 prior line; Part-2: VenDVd in patients RRMM 1–3 prior lines of therapy (no PI)	NCT03314181
CA209-755: randomized trial evaluating nivolumab and daratumumab with or without low-dose cyclophosphamide in patients with RRMM	II	RRMM patients ≥2 prior therapies	Part A: run-in phase + randomization; Part B: randomization; NDC *vs* ND	NCT03184194
				

C, cyclophosphamide; V, bortezomib; R, lenalidomide; D, daratumumab; d, dexamethasone; Ixa, ixazomib; K, carfilzomib; P, pomalidomide; N, nivolumab, Ven, venetoclax; CyBorD, cyclophosphamide–bortezomib–dexamethasone.

### Daratumumab–Lenalidomide–Dexamethasone

DRd regimen was explored in POLLUX trial, a phase 3, randomized, open-label, multicenter study evaluating the safety and efficacy of Rd and DRd in patients with RRMM, with a median of 1 prior treatment line (42). 569 patients with relapsed/refractory MM were randomly assigned to receive Rd with or without daratumumab, each administered until disease progression or unacceptable toxicity. Lenalidomide was given 25 mg PO on days 1 through 21 of each cycle and dexamethasone 40 mg weekly in the Rd arm. In the DRd arm, daratumumab was given at 16 mg/kg IV weekly for 8 weeks in cycles 1 and 2, every 2 weeks for 16 weeks in cycles 3–6, and then every 4 weeks, along with Rd. Safety and efficacy were evaluated after a median follow-up of 54.8 months, with a treatment median duration of 34.3 and 16.0 months in the DRd and Rd groups, respectively (43). PFS in the ITT population for the DRd *vs* Rd groups was 45.0 *vs* 17.5 months (P < 0.0001) respectively, with a 48-month PFS rate of 48% DRd *vs* 21% Rd and an ORR of 93% for DRd (n = 281) *vs* 76% for Rd (n = 276) (P < 0.0001). In patients exposed to one prior treatment line, PFS was 53.3 months in the DRd arm *vs* 19.6 months in the Rd arm (HR 0.42, P < 0.0001). MRD negativity rates (10^−5^) for DRd *vs* Rd were 33 *vs* 7% (P < 0.0001) in the ITT population. Regarding safety profile, grade 3/4 neutropenia was the most relevant hematologic AE, with 57 *vs* 42% in DRd and Rd respectively, followed by anemia and thrombocytopenia (19 *vs* 22% and 15 *vs* 16%, respectively). Non-hematologic AE was dominated by diarrhea 59 *vs* 38% and pneumonia 25 *vs* 17%, respectively in the DRd and Rd arms. Infusion reactions were reported in 48% of patients and were mostly mild; the majority (92%) occurred at the first administration. An updated efficacy and safety data of DRd based on cytogenetic risk status from POLLUX after a median follow-up of 44.3 months showed DRd significantly improved ORR, PFS, and MRD-negativity rates *vs* Rd in patients with both standard and high cytogenetic risk (44). A sub-analysis for elderly patients of POLLUX trials, divided in two groups of 65–74 years and ≥75 years, showed an improvement in PFS, ORR, and MRD-negativity rates for DRd *vs* Rd (45). Regarding safety, hematological AEs were superimposable to other age groups; daratumumab infusion reaction rate was similar in ITT population, but only with 14 and 5% of grade 3/4, respectively, with treatment discontinuation. Overall, in the POLLUX trial, the evidence of the greatest clinical benefit of DRd observed in patients that had received one prior line of therapy supports the use of DRd in patients with RRMM at first relapse. Despite a higher incidence of neutropenia and pneumonia in the DRd arm, treatment discontinuation rate was similar (17 *vs* 15%). PLEIADES is an ongoing, phase 2, non-randomized, multicenter study evaluating the clinical benefit of DRd in RRMM with ≥1 prior line of therapy (46). Daratumumab subcutaneous is administered weekly at 1,800 mg in cycles 1 and 2, then on days 1 and 15 of cycles 3–6, and on day 1 of cycles 7+; lenalidomide at 25 mg PO on days 1–21 of each cycle; dexamethasone: 40 mg PO weekly. An ORR of 93.8%, the primary end-point, was met for the DRd cohort with response rates similar to the POLLUX study. Most common AEs were neutropenia (49%), thrombocytopenia (14%), and pneumonia (12%).

### Daratumumab–Bortezomib–Dexamethasone

The DVd combination was first explored by CASTOR, a phase 3, open-label, randomized, multicenter study evaluating the safety and efficacy of bortezomib–dexamethasone (Vd) alone and plus IV daratumumab (DVd) in 498 patients with RRMM (47). Regarding the administration schedule, in Vd: bortezomib 1.3 mg/m^2^ subcutaneously on days 1, 4, 8, and 11 over each 21-day cycle for eight cycles; dexamethasone 20 mg PO or IV on days 1, 2, 4, 5, 8, 9, 11, and 12 of the eight cycles. In the DVd arm: Vd plus daratumumab 16 mg/kg IV weekly for the first three cycles, once every 3 weeks of cycles 4–8, and every 4 weeks thereafter. Updated results after a median follow-up of 50.2 months showed a median PFS of 16.7 months *vs* 7.1 months (HR: 0.31, P < 0.0001) with DVd and Vd respectively, and regarding patients who received one previous therapy line, the benefit was 27.0 *vs* 7.9 months (HR: 0.21, P < 0.0001) (48). In patients with evaluable response, ORR was 85 *vs* 63% (P < 0.0001), and for those receiving one previous therapy ORR was 92 *vs* 74% (P = 0.0007), respectively. Overall, as in POLLUX, the safety profile of CASTOR trial was marked by a slightly higher incidence in thrombocytopenia and neutropenia, but not translated in a significative rate of discontinuation in the DVd arm *vs* Vd (10 and 9%, respectively). Regarding cytogenetic risk: in high-risk patients, median PFS was 12.6 months with DVd *vs* 6.2 months with Vd (HR: 0.41; P = 0.0106), while in standard cytogenetic risk median PFS was 16.6 *vs* 6.6 months (HR: 0.25; P < 0.0001). Regarding safety profile, most common grade 3/4 hematologic AEs were, for DVd and Vd arm, thrombocytopenia (46 *vs* 33%), anemia (both 16%) and neutropenia (14 *vs* 15%). Non-hematologic AEs comprised mainly of peripheral neuropathy (all grade 50 *vs* 38% for DVd and Vd), upper respiratory tract infections, pneumonia, and hypertension (36 *vs* 18%, 16 *vs* 13%, and 11 *vs* 13%, respectively). Secondary solid or hematological malignancies were reported in 15 (6%) patients who received DVd *vs* four (2%) patients who received Vd. As in POLLUX, also CASTOR trial was analyzed for the elderly population, divided in two groups by age (65 to 74 years and ≥75 years) showing the advantage of DVd over Vd in terms of PFS and ORR of both groups, with a safety profile similar to that of the younger patients (45).

### Daratumumab–Carfilzomib–Dexamethasone

Given the effectiveness of daratumumab with bortezomib-containing regimens, it was also evaluated with the second-generation proteasome-inhibitor carfilzomib (DKd) in the six-arm phase 1b study, proving its efficacy and safety in 85 RRMM patients receiving DKd (49). In each 28-day cycle, daratumumab was administered at 16 mg/kg IV every week on cycles 1**–**2, every 2 weeks on cycles 3**–**6, and every 4 weeks thereafter; carfilzomib was administered weekly on days 1, 8, and 15 of each cycle at 20 mg/m^2^ on day 1-cycle 1 and escalated to 70 mg/m^2^ on day 8-cycle 1; dexamethasone: 40 mg/week. With a median follow-up of 16.6 months, an ORR of 84% was obtained in the whole cohort. Major grade >3 AEs were: thrombocytopenia (31%), anemia (21%), neutropenia (21%), hypertension (18%), and asthenia (12%). Infusion reaction rate was higher when the first infusion of daratumumab was administered as a single dose compared to a split dose (60 *vs* 43%). Updated results after 23.7 months of median follow-up were: ORR was 84%, median PFS was 25.7 months, and median OS was not reached (50). Relevant grade 3/4 AEs were: thrombocytopenia (32%), anemia (21%), neutropenia (21%), hypertension (20%), and upper respiratory tract infections (4%). Multicentric phase 3 CANDOR trial evaluated DKd *vs* Kd allocating in a randomized 2:1 mode to receive DKd or Kd in 28-day cycles until disease progression (51). Carfilzomib was given on days 1, 2, 8, 9, 15, and 16 of each cycle to all patients at 20 mg/m² on days 1 and 2 during cycle 1 and 56 mg/m² thereafter, as IV infusion. Daratumumab (8 mg/kg) was administered as IV infusion on days 1 and 2 of cycle 1 and at 16 mg/kg weekly for the remaining doses of the first two cycles, then every 2 weeks for four cycles (cycles 3 to 6), and every 4 weeks thereafter. Dexamethasone was administered PO or IV at 40 mg weekly (20 mg for patients ≥75 years). A total of 466 patients received either DKd (n = 312) or Kd (n = 154). Median PFS follow-up was 16.9 *vs* 16.3 months, and median PFS was not evaluable and 15.8 months for DKd and Kd, respectively. Treatment with DKd resulted in a 37% reduction in the risk of progression or death (HR, 0.63; P = 0.0027). The ORR in the DKd group was 84 *vs* 75% in the Kd group (P = 0.0080). Severe (grade >3) hematologic and non-hematologic AEs of interest in DKd group *vs* Kd, group were: thrombocytopenia (24 *vs* 18%), respiratory tract infections (27 *vs* 15%), acute renal failure (5 *vs* 7%), cardiac failure (4 *vs* 9%). Updated results of this trial approximately 36 months after the enrollment beginning show a median PFS follow-up was 28.6 *vs* 15.2 months, resulting in a 13.4-month improvement in median PFS which was observed in the DKd arm, with safety data consistent with the previous analysis (52). LYNX is an ongoing, randomized, open-label, multicenter, phase 2 study evaluating the safety and efficacy of DKd (subcutaneous daratumumab) *versus* Kd alone in RRMM patients who were previously exposed to a IV daratumumab-containing therapy, with the scope to evaluate daratumumab retreatment (53, 54). Enrolled patients (expected 230) received one to two prior lines of therapy with at least one prior treatment exposure to daratumumab IV (but not exposed to carfilzomib) and are randomized 1:1 in order to receive DKd or Kd. All patients will receive 28-day cycles of Kd until PD or intolerable toxicity as follows: carfilzomib 20 mg/m^2^ IV on day 1-cycle 1, escalated to 70 mg/m^2^ on days 8 and 15-cycle 1, and 70 mg/m^2^ on days 1, 8, and 15 for each subsequent cycle; dexamethasone 40 mg IV or oral on days 1, 8, 15, and 22 up to cycle 9, then on days 1, 8, and 15 for subsequent cycles. DKd will receive also daratumumab–hyaluronidase 1,800 mg subcutaneous once weekly in cycles 1 and 2, then once every 2 weeks in cycles 3–6, and once monthly for each subsequent cycle. Primary endpoint is rate of ≥VGPR. Exposing again patients to daratumumab, even if by another route of administration, is an attractive opportunity to evaluate how the immune system acts in these conditions and if any kind of immunogenicity could be raised against this monoclonal antibody that potentially could affect a retreatment strategy.

### Daratumumab–Pomalidomide–Dexamethasone

The same trial also evaluated another treatment combination: daratumumab plus pomalidomide–dexamethasone (DPd) (49, 55). Patients in the DPd arm (n = 103) received 28-day cycles of intravenously daratumumab 16 mg/kg (weekly for cycles 1–2, every 2 weeks for cycles 3–6) in combination with pomalidomide 4 mg (on days 1–21) and dexamethasone 40 mg weekly. Among responder patients, ORR was 66%, and the median duration of response was 21.5 months, median PFS was 9.9 months, median OS was 25.1 months with a median follow-up of 28.1 months. Safety profile showed relevant grade >3 AEs as follows: neutropenia (78.6%), anemia (28.2%), thrombocytopenia (19.4%), upper respiratory tract infections (2.9%). MM-0146 is an ongoing, phase 2, non-randomized, multicenter, open-label clinical study evaluating the safety and efficacy of DPd and Pd RRMM patients (N = 112) previously exposed to one or two prior lines of therapy including lenalidomide (56). Patients in the DPd arm will receive 28-day cycles of intravenously daratumumab 16 mg/kg in combination with pomalidomide 4 mg PO daily (days 1–21) and dexamethasone 40 or 20 mg/day, depending on age, on days 1, 8, 15, and 22. Daratumumab was administered on cycles 1–2 weekly, twice weekly for cycles 3–6, and every 4 weeks thereafter. After a median follow-up of 17.2 months, in the ITT population (N = 112), ORR was 77.7%, PFS was not reached. Safety analysis reported that the most common grade 3/4 AEs were neutropenia (62.5%), anemia (17.9%), and pneumonia (13.4%). RRMM patients undergoing a daratumumab-containing regimen are often previously exposed to IMIDs. Therefore, effectiveness of pomalidomide in overcoming IMID resistance could potentially enhanced by daratumumab co-administration, giving a new chance to use also IMID activity on myeloma cells.

### Daratumumab–Cyclophosphamide–Dexamethasone

The alkylating agent cyclophosphamide was challenged with daratumumab in different modalities. A phase II clinical trial enrolling 120 patients with RRMM who had received at least one line of prior therapy randomize patients in two arms. In the A arm patients receive daratumumab, weekly low dose of cyclophosphamide, dexamethasone, and pomalidomide (DCdP); in the B arm patients receive DCd and pomalidomide only at progression of disease (57). In the DCdP arm patients were randomized to receive daratumumab 16 mg/kg weekly cycles 1–2, every 2 weeks cycles 3 to 6, monthly on cycle 7 and beyond: dexamethasone 40 mg PO weekly, cyclophosphamide 400 mg PO weekly and pomalidomide 4 mg PO days 1–21 of 28-day cycles. In the DCd arm patients received daratumumab, cyclophosphamide, and dexamethasone at the same dose; pomalidomide was added after proof of disease progression. After a median of 8.2 months, ORR in the DCdP arm was 88.5% compared with 50.8% for DCd arm, and PFS was not reached for the DCdP arm. Incidence of grade 3/4 hematologic toxicities included a high incidence of neutropenia 74 *vs* 30%, and thrombocytopenia was 4.9 and 13.6% in DCdp *vs* DCdP, respectively. Infectious AEs were: febrile neutropenia was 8.2 *vs* 6.8% and pneumonia 18 *vs* 16.9%, respectively. Daratumumab was also evaluated with cyclophosphamide–bortezomib–dexamethasone (DCyBorD) in a small number of patients. LYRA, an ongoing, phase 2, single-arm, open-label, multicenter study evaluates the safety and efficacy of this regimen either for the treatment of MM in patients who have not received previous treatment and for one RRMM of one treatment line (n = 14) (58, 59). Daratumumab was administered at 8 mg/kg intravenously on days 1–2 of cycle 1, then 16 mg/kg weekly in cycle 1 (day 8) and cycle 2, then twice weekly in cycles 3–6, then every 4 weeks in cycles 7–8. Cyclophosphamide was given as 300 mg/m^2^ PO weekly on days 1, 8, 15, and 22 of each cycle; bortezomib at 1.5 mg/m^2^ subcutaneously weekly on days 1, 8, and 15; dexamethasone: 40 mg weekly. With a median follow-up of 26.6 months, 79% of RMM patients obtained ORR, and median PFS was not reached (60). In RRMM patients, hematological and non-hematological grade >3 relevant reported AEs were: neutropenia (21%) and diarrhea (7%).

### Daratumumab–Venetoclax–Dexamethasone

The BCL-2 inhibitor venetoclax, largely adopted in other lymphoproliferative disorders, is on evaluation in a phase 1/2 trial also in patient with RRMM with and without t(11;14) (61). Venetoclax is given in combination with daratumumab and dexamethasone with or without bortezomib (VenDd or VenDVd) and patients (n = 48) are divided in two cohorts of patients, depending on t(11;14) status. With a median follow-up of 10 and 9 months for VenDd and VenDVd respectively, ORR was 96 and 92%, and median PFS was not reached. Grade ≥3 AEs were neutropenia (17%), hypertension (12%), fatigue and hyperglycemia (both 8%) for patients on VenDd, and insomnia (21%), diarrhea and thrombocytopenia (both 8%) for patients on VenDVd. A phase 1/2 study enrolling RRMM patients is designed to administer DVd with or without venetoclax and evaluate MRD rates and the role of t(11;14) as marker of disease (62).

### Daratumumab–Nivolumab/Daratumumab–Nivolumab–Cyclophosphamide

Anti-PD1 nivolumab is another molecule with a promising anti-myeloma activity, as shown by two ongoing trials. CA209-755, an ongoing phase 2, randomized, multicenter study, is expected to enroll 60 patients with RRMM receiving daratumumab**–**nivolumab with or without cyclophosphamide (DN *vs* DNc) (63). In a 28-day cycle: daratumumab IV weekly is given 16 mg/kg for cycles 1**–**2, then every 2 weeks for cycles 3**–**6, then every 4 weeks thereafter; nivolumab 240 mg IV every 2 weeks in cycles 1**–**6 and 480 mg weekly subsequently. When added, cyclophosphamide was given 50 mg orally once daily on days 1**–**28. A total of 40 patients were randomized in two consecutive phases and after a median follow-up of 8.6 months: ORR (>SD) was 85 and 80% for DN and DNc, respectively. Most relevant toxicity was infections. CA209-039 is another phase 1/2 ongoing trial investigating the role of nivolumab in several hematological neoplasm, RRMM included, as monotherapy or in combination regimens across various associations (64). Patients with RRMM are being assigned to one of the following arms: daratumumab + nivolumab or daratumumab + nivolumab + pomalidomide and dexamethasone. The aim of the trial is to evaluate the safety of these combinations. A limitation of this trial is that nivolumab is not challenged with another agent that is commonly adopted in combination in clinical practice (bortezomib, lenalidomide), but only with cyclophosphamide.

### Daratumumab–Durvalumab

The human monoclonal antibody anti-PD-L1 durvalumab, already adopted in lung neoplasm, is currently being tested in MEDI4736-MM-003, a safety and efficacy trial of daratumumab IV when administered in combination with daratumumab (DD) for the treatment of RRMM (65). The study will also conduct a preliminary analysis of the addition of pomalidomide and low-dose dexamethasone to DD either in patients with progressive disease with DD or as upfront therapy. Daratumumab is also under evaluation with another humanized monoclonal antibody anti-PD-L1, atezolizumab, in GO29695 trial (66, 67). This phase 1b, open-label, non-randomized, multicenter study is expected to enroll approximately 300 patients exposed to different drug combinations. Three arms are planned: daratumumab–atezolizumab (DA) alone (explored in a run-in and expansion phases), DA–lenalidomide, DA–pomalidomide. In a 28-day cycle, daratumumab and atezolizumab are administered intravenously at 16 mg/kg and 840 mg, respectively, lenalidomide and pomalidomide at different dosages. A total of 24 patients were enrolled in the study and treated; ORR was 67% in the DA (run-in phase) cohort, 57% in the DA + lenalidomide (dose escalation) cohort, and 67% (n = 4) in the DA + pomalidomide (dose escalation phase) cohort. Regarding AEs, grades 3–4 occurred in 33% of patients in the DA (run-in phase) cohort, 75% in the DA (expansion phase), 86% in the DA + lenalidomide (dose escalation phase) cohort and 100% of DA + pomalidomide (dose escalation phase).

### Daratumumab–Ixazomib

Finally, the new generation oral PI ixazomib was evaluated with daratumumab and dexamethasone as interim efficacy analysis of the phase 2 trial, without published results at the moment (68).

## Toxicity Profile

Daratumumab generally shows a favorable toxicity profile with easily manageable AEs. Being part of the anti-myeloma monoclonal antibody class, daratumumab mostly shows AE and a toxicity profile commonly found in this category of compounds (es. elotuzumab). In clinical practice, a relevant topic when using daratumumab, and generally monoclonal Abs, is the infusion-related reactions (IRRs). In the SIRIUS trial, single agent daratumumab had a 45% IRR rate, represented by respiratory symptoms, such as nasal congestion, rhinitis cough, throat irritation, and dyspnea, mostly grades 1–2 (40). IRRs are characterized by a typical onset timing: they usually occur with maximum incidence at first infusion (96%) or, at least, at the second one, but with lower incidence (7%). The same IRR rate and timing of onset is found also when daratumumab is combined with other anti-myeloma agents. In the CASTOR trial, DVd treatment is associated with an IRR rate of 45%, with almost all events occurring during the first administration (47). Moreover, in the POLLUX trial, a 48% of IRRs were reported for daratumumab when infused as combined regimen DRd, a 50% in MMY1001 when daratumumab is administered as DPd (42, 69). Overall, IRRs are easily both preventable and manageable with adequate pre- and post-medications as antihistamines, corticosteroids, montelukast acetaminophen as well as interrupting and slowing down the infusion rate of daratumumab (70). Minimizing the possibility of IRR by a slow rate of intravenous infusion of daratumumab is routinely adopted but is also time-consuming: 7 h at first–second administration to 3 h subsequently. A way to possibly reduce the IRR rate together with a faster administration modality is subcutaneous injection, as it was explored by COLUMBA trial (71). This multicenter, open-label, non-inferior, randomized, phase 3 trial showed that in RRMM patients, a 1,800 mg subcutaneous flat dose of daratumumab delivered in 5 min is not inferior in terms of efficacy compared to the intravenous route. With the limitation of a non-blinded trial (for both patients and clinicians), grade 3 IRR occurred in 2% of patients, and no grade 4 or 5 IRRs in the subcutaneous group were reported. As reported about the safety profiles of the trials cited in this review, other common ADRs are mostly hematological or related to hematological toxicity such as anemia, neutropenia, thrombocytopenia, fatigue, pyrexia, pneumonia, and upper respiratory tract infections. It is predictable and intuitable that these types of AEs are notably influenced by the daratumumab-associated anti-myeloma agent in a certain regimen adopted. A combined analysis of five phase III randomized controlled trials showed that relative risk of all grades of neutropenia and leukopenia in patients undergoing daratumumab-based regimens was higher than that in the control arms despite lower RR of anemia (72). Finally, not properly definable as toxicity or AD, daratumumab can affect the indirect Coombs test when performed as blood group compatibility test, due to the expression of its target CD38 on red blood cells (73).

## Conclusions

The effectiveness and the favorable toxicity profile of daratumumab for the treatment of both NDMM and RRMM have led to a wide spreading of the use of this new immunotherapeutic agent alone and in combination with standard of care anti-MM treatment. Emerging data from clinical trials are crucial to define newer possible treatment combination since combination treatments involving molecules with different therapeutic target on myeloma cells. The improvement rates of CR with the adoption of novel drugs are nowadays to be considered as a chronic disease relapse eventually appears along the clinical history of almost each patient. Simultaneously, the disease refractoriness to a specific class of drug is a concerning issue for clinicians. The advent of daratumumab, anti-CD38 antibody, gave to physicians one more effective molecule to treat this through the phase of the clinical course of MM. The toxicity profile of daratumumab is also favorable and easily manageable by clinicians. Ongoing trials are giving the opportunity of exploring its effectiveness also combined with other mechanism of actions, such as cyclophosphamide, venetoclax, and molecules acting on the PD-1 pathway. Given the effectiveness of daratumumab combination and its safety profile still adopted in clinical practice, efforts are mandatory to conduct these (and future) trials to explore other combinations.

## Author Contributions

Conceptualization, methodology, design, and writing: MO, LC, SM, DN, GM, AO, and CC. Supervision: MO, GM, AO, and CC. All authors contributed to the article and approved the submitted version.

## Conflict of Interest

The authors declare that the research was conducted in the absence of any commercial or financial relationships that could be construed as a potential conflict of interest.
